# Mass Balance of Perfluoroalkyl Acids, Including Trifluoroacetic Acid, in a Freshwater Lake

**DOI:** 10.1021/acs.est.1c04472

**Published:** 2021-12-20

**Authors:** Maria K. Björnsdotter, Leo W. Y. Yeung, Anna Kärrman, Ingrid Ericson Jogsten

**Affiliations:** Man-Technology-Environment Research Centre (MTM), Örebro University, 701 82 Örebro, Sweden

**Keywords:** ultrashort-chain perfluoroalkyl acids, global radiation, atmospheric oxidation, atmospheric deposition, flux, precursors

## Abstract

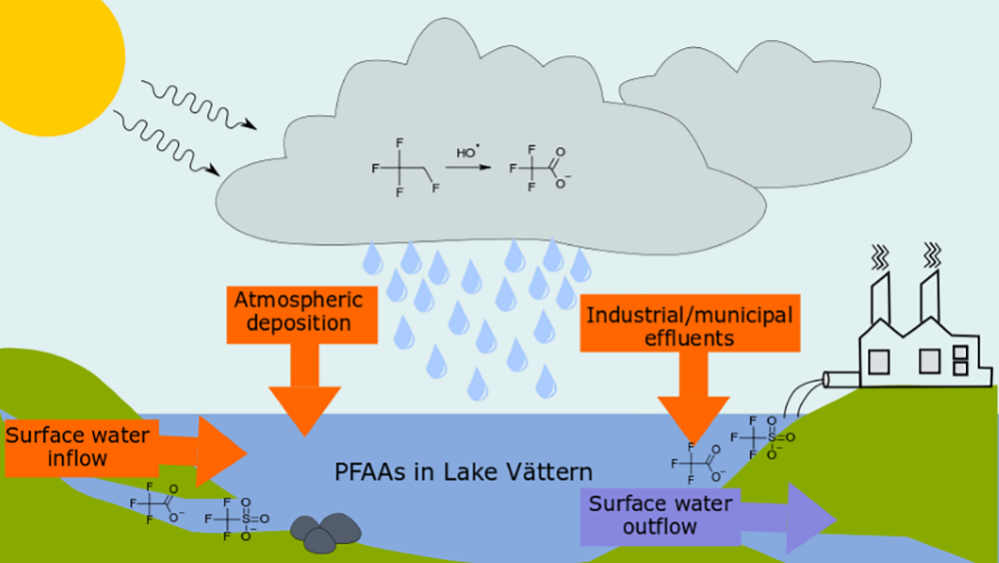

Perfluoroalkyl acids (PFAAs) are highly persistent chemicals that are ubiquitously found in the environment. The atmospheric degradation of precursor compounds has been identified as a source of PFAAs and might be an important pathway for contamination. Lake Vättern is one of Sweden’s largest lakes and is an important source for drinking water. In addition to contamination via atmospheric deposition, the lake is subject to several potential contamination sources via surface water inflow. The relevance of different sources is not well understood. A mass balance of selected PFAAs was assembled based on measured concentrations in atmospheric deposition, surface water from streams that constitute the main inflow and outflow, and surface water in the lake. The largest input was seen for trifluoroacetic acid (150 kg/year), perfluoropropanoic acid (1.6 kg/year), perfluorobutanoic acid (4.0 kg/year), and perfluoro-octanoic acid (1.5 kg/year). Both atmospheric deposition and surface water inflow was found to be important input pathways. There was a positive correlation between the input of most perfluoroalkyl carboxylic acids via atmospheric deposition and global radiation and between the input via surface water inflow and catchment area. These findings highlight the importance of atmospheric oxidation of volatile precursor compounds for contamination in surface waters.

## Abstract 2

Atmospheric oxidation of volatile precursors is a major source of perfluoroalkyl carboxylic acids to a freshwater lake via atmospheric deposition.

## Introduction

Perfluoroalkyl acids (PFAAs) are man-made chemicals that have been produced since the 1950s, and their widespread use has resulted in environmental pollution worldwide. Their amphiphilic properties make PFAAs useful in a wide range of applications. The use of PFAA-containing aqueous film-forming foam (AFFF) for firefighting training has been identified as a major source of groundwater contamination.^1^ PFAAs can also be formed in the environment by the transformation of precursor compounds,^2−4^ which may undergo long-range transport before depositing through atmospheric deposition. Atmospheric deposition was found to be a dominant pathway of PFAAs to the Baltic Sea.^5^

PFAAs include perfluoroalkyl carboxylic acids (PFCAs) and perfluoroalkyl sulfonic acids (PFSAs), which are divided into short- and long-chain compounds depending on the length of their carbon backbone. Long-chain PFCAs and PFSAs are defined as those containing at least eight and six carbon atoms or more, respectively, and have a greater potential for bioaccumulation.^6^ The much less-studied ultrashort-chain PFAAs are defined as the PFCAs and PFSAs with a chain length of 2–3 and 1–3 carbon atoms, respectively. These include trifluoroacetic acid (TFA), perfluoropropanoic acid (PFPrA), trifluoromethane sulfonic acid (TFMS), perfluoroethane sulfonic acid (PFEtS), and perfluoropropane sulfonic acid (PFPrS).

Increased attention to TFA was paid in the late 90s after the implementation of the Montreal Protocol in 1989, which aimed at phasing out ozone-depleting chlorofluorocarbons that were used as refrigerants. This resulted in the introduction of hydrofluorocarbons (HFCs) and hydrochlorofluorocarbons (HCFCs). The atmospheric degradation of HFCs and HCFCs results in the formation of TFA^7^ and has been identified as a major source of TFA to the environment.^8^ A recent study on ice cores shows an increased deposition of TFA, PFPrA, and perfluorobutanoic acid (PFBA) since 1990^9^ and that the environmental levels of TFA are expected to increase in the future as a result of a shift from HFCs toward hydrofluoroolefins (HFOs).^10^

TFA is a substance of multiple sources, and high concentrations have been observed downstream a chemical plant,^11^ at firefighting training sites, and in landfill leachate.^12^ The sources and environmental occurrence of ultrashort-chain PFAAs are not as fully documented as those of their longer chain homologs. While the occurrence of TFA has been frequently documented in surface waters, only a few studies have included other ultrashort-chain PFAAs. Ultrashort-chain PFAAs have been reported in surface water samples collected in connection to potential point sources such as landfills, military training sites, and waste management facilities.^12,13^ PFPrA has been reported in atmospheric precipitation^14^ and has been found in Arctic ice cores.^9^ TFMS was recently reported in the environment for the first time^15^ and also in surface water and groundwater far away from primary environmental emission points.^16^ PFEtS and PFPrS have been reported in municipal and industrial wastewater.^17^ The potential sources and the environmental fate of these substances are not yet well understood.

Ultrashort-chain PFAAs are highly polar, and the potential for bioaccumulation in biota is low. However, accumulation in plants has been shown.^18,19^ There are limited data available on human exposure to ultrashort-chain PFAAs, but TFA was recently detected in human blood.^20^ The high polarity makes ultrashort-chain PFAAs mobile in the environment, and accumulation in aquatic bodies has been observed.^21,22^ Their high persistence, together with their mobility in the environment, makes them a potential threat to drinking water, and it may be challenging and costly to remove contamination once adverse effects have been identified. Efforts have been made to remove PFAAs from drinking water due to the widespread contamination of groundwater; however, the effectiveness of commonly used technologies, such as granular activated carbon, decreases with shorter carbon chain length, and the smallest and most polar molecules are not removed at all.^11,23^

There is a need to understand the occurrence and mass flows of ultrashort-chain PFAAs to assess the environmental hazard and potential for human health effects. The environmental fate and behavior of pollutants can be better understood by multimedia mass-balance models taking sources, emissions, mass transport, and chemical characteristics into consideration.^24^ Field monitoring should thus aim for assessing less-studied chemicals in defined environmental systems to facilitate increased environmental understanding and decision-making. The present study includes input and output calculations of ultrashort-, short-, and long-chain PFAAs for one fresh water lake in Sweden (area, 1900 km^2^; volume, 74 km^3^) that is a drinking water source for almost 300,000 people and is subject to anthropogenic pressures such as wastewater discharges, landfill leachates, and agricultural and industrial activity. In addition, two airports are located in the catchment area, with elevated concentrations of PFAAs in both groundwater and surface water as a result.^25^ Occurrence of PFAAs in fish from the lake has previously been reported.^26^ The relative contribution of PFAA input from different sources, including local sources and atmospheric deposition, is not yet fully understood, and appropriate management strategies of this drinking water reservoir is lacking. In the field of PFAAs, most studies have focused on short- and long-chain PFAAs. While the environmental occurrence of TFA has been well documented in the past, there is little published data on other ultrashort-chain PFAAs, and the relevance of different sources has not been investigated, including for TFA.

The aim of the present study was to assess the relevance of local and diffuse sources of PFAAs to a fresh water lake. A mass balance assessment was performed for 14 PFAAs, including the ultrashort-chain PFAAs TFA, PFPrA, and TFMS based on measured inputs (surface water inflow, atmospheric deposition, and effluents from a sewage treatment plant and a paper mill), outputs (surface water outflow), and the inventory of PFAAs in the lake. The correlation between the input from atmospheric deposition and global radiation was examined to elucidate the relevance of the atmospheric degradation of volatile precursors as a pathway for PFAAs to the lake.

## Materials and Methods

### Chemicals and Reagents

Detailed information about chemicals and reagents is provided in the Supplementary Information.

### Sample Collection

Bulk atmospheric deposition samples (*n* = 12) were collected on an island in Lake Vättern over 1 month of sampling time from July 2018 to June 2019. Open sampling containers were used to collect both wet and dry deposition. Deposition samples (*n* = 9) were collected using polyethylene funnels (diameter, 248 mm) connected to polyethylene containers. Snow samples (*n* = 3) were collected in polypropylene boxes from December 2018 to February 2019. Surface water samples (grab samples) were collected from 19 streams (*n* = 76) at four occasions from March to December 2019, representing four different seasons. Of the 19 streams, 17 streams were chosen as they represent the relevant surface waters that discharge into Lake Vättern: one stream represents the main outflow, and another was included as it passes near an airport with known PFAA contamination. The latter was not included in the mass balance as it discharges into another stream (included in the mass balance) that discharges into Lake Vättern. The streams included in the study are subject to different anthropogenic pressures including sewage treatment plants, landfills, industries, firefighting training sites, and agriculture as well as stormwater from polluted areas (some with known PFAA pollution). Reference samples (*n* = 4) were collected at four occasions from March to December 2019 from a stream upstream from the study area and downstream from a lake that is not a recipient of any known local contamination. Effluent water samples (24 h flow proportional composite samples) were collected from a sewage treatment plant (*n* = 4) and a paper mill (*n* = 4) that discharges directly into Lake Vättern at four occasions from March to December 2019. Other sewage treatment plants and industries discharge into streams, which are included in the monitoring. Surface water samples (grab samples) were collected from two locations in Lake Vättern during spring and summer 2019. Samples were collected at 0.5 m depth in April when the water column is mixed (*n* = 2) and at 0.5 and 30 m depths in July or August when the lake is stratified (*n* = 4). Information about the lake mixing regime was obtained by oral communication with the water management association Vätternvårdsförbundet. The sampling locations are shown in Figure S1, and the potential contamination sources, catchment areas, and flow rates of respective streams are listed in Table S1.

All sample containers were precleaned with ultrapure water followed by methanol. The surface water, lake water, and effluent samples were collected in precleaned polyethylene containers that were rinsed three times with the sample matrix before collecting the sample. The samples were sealed and transported to the laboratory. Snow samples were melted and transferred to precleaned polyethylene containers. All samples were stored at 4 °C until processing.

### Sample Preparation and Analysis

All samples were ultrasonicated for 10 min to desorb target analytes that possibly adhere to the inner surface of the containers. The containers were rinsed with methanol once the sample was taken out, and methanol was combined with the sample.

Surface water samples and effluent from the sewage treatment plant and the paper mill were filtered with glass microfiber filters (1.2 μm) prior to extraction to prevent clogging of the solid-phase extraction sorbent. The filters were extracted three times with methanol by ultrasonication for 30 min followed by centrifugation at 7100*g* for 5 min. The filter extract was combined with the water sample to obtain results for both dissolved and sorbed PFAA. Atmospheric deposition samples were not filtered prior to extraction since the amount of particles were low. The pH was adjusted to 4 in all samples by the addition of acetic acid prior to extraction. Atmospheric deposition (200 mL), surface water from inflowing streams (500 mL), surface water from Lake Vättern (1000 mL), and effluent from the sewage treatment plant and the paper mill samples (500 mL) were extracted by weak anion exchange solid-phase extraction following the ISO25101 method with some modifications (details are provided in the SI).

A novel analytical technique using supercritical fluid chromatography (SFC) coupled with tandem mass spectrometry (MS/MS) (Acquity Ultra Performance Convergence Chromatograph and Xevo TQ-S Micro, Waters Corporation, Milford, MA, USA) operated in negative electrospray ionization mode was used for the separation and quantification of C_1_–C_4_ PFAAs after some modifications for improved separation of ultrashort-chain PFAAs, including TFMS.^27^ An SFC Torus DIOL column (3.0 mm id, 150 mm length, 1.7 μm particle size) (Waters Corporation, Milford, MA, USA) maintained at 50 °C was used to achieve chromatographic separation. Separation and quantification of C_5_–C_12_ PFAAs were performed using ultraperformance liquid chromatography (UPLC) MS/MS (Acquity Ultra Performance Liquid Chromatograph and Xevo TQ-S, Waters Corporation, Milford, MA, USA) operated in negative electrospray ionization mode. A UPLC BEH C18 column (2.1 mm id, 100 mm length, 1.7 μm particle size) (Waters Corporation, Milford, MA, USA) maintained at 50 °C was used to achieve chromatographic separation. Detailed information about the SFC, UPLC, and source parameters is provided in the SI. At least two MS/MS transitions were monitored for each target analyte except for TFA, PFPrA, PFBA, and PFPeA, where only one transition was monitored. MRM transitions for all target analytes are provided in Table S2.

### Quality Assurance and Quality Control

Isotope dilution was used for quantification using mass-labeled internal standards that were added prior to extraction. Corresponding mass-labeled internal standards were available for most target analytes, including for TFA. Mass-labeled PFBA and PFBS were used for quantification of PFPrA and TFMS, respectively. The repeatability of the method was evaluated based on the relative standard deviation (RSD) of fortified samples (*n* = 11) at a concentration of 1 ng per 250 mL sample. The RSD was in the range of 11–17%. Extraction efficiencies were assessed based on the peak area of native standards spiked to test samples (*n* = 11) after subtraction of the background concentrations in the samples. The extraction efficiency was in the range of 58–123%. Mass-labeled recovery standards were added to the sample extracts prior to injection to monitor the recovery of the method. The methanol used was checked for contamination, and procedural blanks were included in each batch of samples. None of the target analytes were observed in the methanol. Limits of detection (LOD) were calculated as the average concentration in repeated procedural blank extractions plus three times the standard deviation for samples of atmospheric deposition (*n* = 8) and for samples of surface water, lake water, and effluents (*n* = 8). For those analytes that were not observed in procedural blanks, the LOD was set as the lowest calibration point with a signal-to-noise ratio of at least 3. Field blanks for surface water (*n* = 4) and rain (*n* = 2) were included to ensure that no contamination occurred during sampling. The field blanks were sampling containers with ultrapure water that were opened during sampling, closed, and transported back to the laboratory and treated in the same way as the samples. None of the target analytes were observed in the field blanks. Detailed information about repeatability, extraction efficiencies, and LODs are provided in Table S3.

### Statistical Analysis

In total, 22 PFAA were analyzed, but only compounds that were detected in more than half of the samples (detection frequency > 50%) were included in the statistical analysis and subsequently reported. Spearman rank correlations between the PFAA input via atmospheric deposition and global radiation and between the PFAA input via inflowing surface water and catchment area were calculated.

### Input Pathways

#### Surface Water Inflow

The input from surface water inflow, *N*_streams_ (kg/year), was calculated based on the median concentration in the surface water (kg/m^3^) and the flow rate per year (m^3^/year). Low-bound estimates (LBE) and high-bound estimates (HBE) were calculated based on the lowest and highest concentrations measured, respectively. The input from surface water inflow was calculated according to
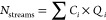
where *C_i_* is the PFAA concentration (kg/m^3^) in stream *i* and *Q_i_* is the flow rate per year (m^3^/year) of stream *i*. The flow rate per year was retrieved from a database created by the Swedish Meteorological and Hydrological Institute and the Swedish Agency for Marine and Water Management and is based on measured data (*n* = 3) and model calculations using the Hydrological Predictions for the Environment (HYPE) model (*n* = 12) (Table S1). Data was not available for two streams, and the flow rate per year was calculated based on flow rate measurements at four occasions during March to December 2019 and calibrated by linear calibration against the modeled flow rate per year from an adjacent catchment area.

#### Atmospheric Deposition

The input from atmospheric deposition, *N*_deposition_ (kg/year), was calculated based on the measured concentration in atmospheric deposition (kg/m^3^) in monthly samples and the amount of atmospheric deposition over Lake Vättern per month (m^3^/month). The input from atmospheric deposition was calculated according to
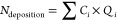
where *C_i_* is the PFAA concentration (kg/m^3^) in atmospheric deposition collected during month *i* and *Q_i_* is the amount of atmospheric deposition (m^3^) over Lake Vättern during month *i*. The amount of atmospheric deposition is based on measurements by the Swedish Meteorological and Hydrological Institute at a meteorological station on the same island as the precipitation samples were collected (Table S4). The input from atmospheric deposition rests on the assumption that the amount of atmospheric deposition is representative for the entire surface area of Lake Vättern.

#### Effluents from the Sewage Treatment Plant and Paper Mill

The input from effluents from the sewage treatment plant and the paper mill, *N*_effluent_ (kg/year), was calculated based on the median concentration in the effluent (kg/m^3^) and the flow rate per year (m^3^/year). The LBE and HBE were calculated based on the lowest and highest concentrations measured, respectively.
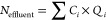
Here, *C_i_* is the PFAA concentration (kg/m^3^) in effluent *i* and *Q_i_* is the flow rate per year (m^3^/year) of effluent *i*.

### Output Via Surface Water Outflow

The output from surface water outflow, *N*_outflow_ (kg/year), was calculated based on the median concentration (kg/m^3^) in the stream that represents the main outflow and the flow rate per year (m^3^/year). LBE and HBE were calculated based on the lowest and highest concentrations measured, respectively. The output from surface water outflow was calculated according to
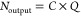
where *C* is the PFAA concentration (kg/m^3^) in the outflowing stream and *Q* is the flow rate per year (m^3^/year). The flow rate per year was collected from a database created by the Swedish Meteorological and Hydrological Institute and the Swedish Agency for Marine and Water Management and is based on measured data.

Other output pathways such as transformation of PFAAs in water, volatilization, evaporation, groundwater recharge, and sediment burial were not considered. Annual evaporation from the lake is provided in Table S4. Transformation in water was estimated to account for less than 0.5% of the output for PFHxA, PFOA, PFDA, and PFOS in the Baltic Sea.^5^ Sediment burial is not thought to be relevant for ultrashort-chain and short-chain PFAAs but might be relevant for long-chain PFAAs. Sediment burial has been estimated to account for 1.5% (PFHxA), 3.6% (PFOA), 26–32% (PFDA), and 9.5% (PFOS) of the output in the Baltic Sea.^5^

### Inventory

The inventory in Lake Vättern, *M*_water_ (kg), was calculated based on the average concentration in the water basin (kg/m^3^) and the volume of water in the water basin (m^3^). The concentration in the water basin was calculated according to
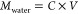
where *C* is the PFAA concentration (kg/m^3^) in the water basin and *V* is the volume of water in the water basin (m^3^).

## Results and Discussion

Detailed information about the PFAA concentrations is provided in Tables S5–S15. TFA was the most abundant PFAA in both atmospheric deposition and in surface water and was detected in all atmospheric deposition samples analyzed at concentrations ranging from 18 ng/L in January 2019 to 300 ng/L in April 2019. The monthly deposition flux of TFA was in the range from 0.30 μg/m^2^ in January 2019 to 16 μg/m^2^ in July 2018. The annual deposition flux of TFA, calculated as the sum of the monthly deposition fluxes, was 52 μg/m^2^. This is approximately four times lower than the annual deposition flux of TFA reported in Germany in 2018.^28^ PFPrA was the third most abundant PFAA in atmospheric deposition (after PFBA) and was detected in all atmospheric deposition samples analyzed at concentrations ranging from 0.92 ng/L in December 2019 to 3.7 ng/L in April 2019. The monthly deposition flux of PFPrA was in the range from 0.02 μg/m^2^ in January 2019 to 0.26 μg/m^2^ in July 2018. The annual deposition flux of PFPrA was 0.78 μg/m^2^. An earlier study reported annual fluxes of 20 PFAAs, including PFPrA, in two locations in Japan and in the United States during 2006–2007 and 2007–2008.^14^ PFPrA was the only ultrashort-chain PFAA included and was the most dominant compound in samples from both countries. Among the ultrashort-chain PFSAs, only TFMS was detected in two atmospheric deposition samples in the present study in the concentration range 0.13–0.15 ng/L. The deposition flux of TFMS was 0.007–0.01 μg/m^2^ (annual deposition flux, 0.02 μg/m^2^). To the best of our knowledge, the presence of TFMS in atmospheric deposition samples is reported here for the first time. PFAAs in precipitation samples have been documented in samples from remote and rural locations,^5,14,29−32^ but only a limited number of studies have reported the occurrence of ultrashort-chain PFAAs.

In surface water from streams, TFA was detected in 91% of the samples (*n* = 79) at concentrations ranging from 30 ng/L to 820 ng/L. The measured TFA concentration in most of the samples was in the same range as previously reported in rivers in China^33,34^ but below the concentrations recently reported in rivers in Germany.^11,35^ PFPrA, TFMS, PFEtS, and PFPrS were detected in 73, 91, 42, and 38% of the samples, respectively, at concentrations in the range 0.60–2.9 ng/L (PFPrA), 0.11–15 ng/L (TFMS), 0.24–0.62 ng/L (PFEtS), and 0.44–3.5 ng/L (PFPrS).

Regarding the inventory of Lake Vättern, most PFAAs were detected in most of the samples. Similar concentrations were detected at both locations, and there was no difference between samples collected in spring compared to summer or between samples collected at 0.5 and 30 m in the summer when the lake was stratified (4–26% relative standard deviation). The measured concentrations are provided in Table S15 in the SI. Among the ultrashort-chain PFAAs, TFA, PFPrA, and TFMS were detected in the lake at concentrations 34 ± 5.2, 0.50 ± 0.04, and 0.26 ± 0.07 ng/L, respectively. PFEtS and PFPrS were not detected in the lake. TFA has been reported in surface water from rivers at various concentrations up to 140 μg/L, but there are limited data on TFA concentrations in lakes. A few studies have reported TFA concentrations in the range 6.8–800 ng/L in lake samples from China collected in 2001 and 2012,^33,34^ and one study reported concentrations of TFA in surface water from the Great Lakes in the range of 55–315 ng/L in samples collected in 1998 and 2000.^36^ In a Nordic screening study, PFPrA could be detected in two locations at similar concentrations.^37^ However, PFPrA could not be detected in Lake Vättern (<0.2 ng/L) in surface water from 2017, suggesting a buildup in concentration due to the annual input from atmospheric sources. To our knowledge, there are no previous studies reporting concentrations of TFMS in surface water in lakes. Other studies have reported ultrashort-chain PFAAs in other matrices such as wastewater,^17^ tap water,^38^ and bottled water, where PFPrA was found to constitute up to 42% of total PFAAs.^39^

The concentrations of short- and long-chain PFAAs in surface water samples from the lake are similar to concentrations across the Great Lakes between 2008 and 2017^32^ but much lower in comparison to concentrations in Tangxun Lake, China (sum PFAAs, 4570–11,900 ng/L), in 2011.^40^ Short-chain PFAAs accounted for the majority of PFAA contamination (PFBS, 3660 ng/L; PFBA, 4770 ng/L) in samples from Tangxun Lake. Ultrashort-chain PFAAs were not included in that study.

The results of the mass balance of PFAAs in Lake Vättern are summarized in Table 1 and illustrated in Figure S2. Only target analytes with a detection frequency of at least 50% in atmospheric deposition or in surface water were included in the mass balance, and data below the LOD was treated as LOD/2. The input from the sewage treatment plant and the paper mill that discharged directly into the lake was not reported separately but was combined and reported together with the input from surface water inflow. This was done because the input from the sampled sewage treatment plant and the paper mill was not representative for those sources to the lake, since some of the streams receive effluent from industries and sewage treatment plants, and thus, input from effluents like these is already included in the input from streams.

**Table 1:** Summary of PFAA Mass Balance in Lake Vättern Based on PFAA Inventory (kg), Input from Surface Water Inflow, Effluents, Atmospheric Deposition (kg/year), and Output Via Surface Water Outflow (kg/year)^a^

	inventory	input (kg/year)			output (kg/year)	
	water column (kg)	surface water inflow and effluents	atmospheric deposition	total input	surface water outflow	input–output (kg/year)
TFA	2600	74 (45–86)	98	170 (140–180)	24 (17–28)	150 (120–170)
PFPrA	38	0.48 (0.30–0.88)	1.5	2.0 (1.8–2.4)	0.39 (0.36–0.54)	1.6 (1.3–2.0)
PFBA	42	1.1 (0.51–1.5)	3.3	4.4 (3.8–4.8)	0.36 (0.03–1.3)	4.0 (2.5–4.8)
PFPeA	34	0.14 (0.04–1.2)	0.27	0.41 (0.31–1.5)	0.02 (0.02–0.55)	0.39 (−0.24–1.5)
PFHxA	45	0.47 (0.08–0.95)	0.53	1.0 (0.61–1.5)	0.35 (0.30–0.39)	0.65 (0.22–1.2)
PFHpA	30	0.50 (0.17–0.81)	0.51	1.0 (0.68–1.3)	0.23 (0.22–0.28)	0.78 (0.40–1.1)
PFOA	140	1.6 (1.2–2.0)	1.5	3.1 (2.7–3.5)	1.6 (1.1–1.9)	1.5 (0.82–2.4)
PFNA	15	0.15 (0.04–0.23)	0.31	0.46 (0.35–0.54)	0.12 (<0.01–0.23)	0.34 (0.12–0.54)
PFDA	2.3	0.03 (0.01–0.09)	0.16	0.19 (0.17–0.25)	0.01 (0.01–0.05)	0.18 (0.12–0.24)
PFUnDA	n.d.	0.03 (0.03–0.04)	0.10	0.13 (0.13–0.14)	0.03 (0.03–0.03)	0.10 (0.10–0.11)
TFMS	20	0.51 (0.24–0.89)	0.03	0.54 (0.27–0.92)	0.20 (0.12–0.37)	0.34 (−0.10–0.80)
PFBS	17	0.27 (0.18–0.39)	0.06	0.33 (0.24–0.45)	0.15 (0.12–0.16)	0.18 (0.08–0.33)
PFHxS	52	0.29 (0.23–0.43)	0.06	0.35 (0.29–0.49)	0.38 (0.35–0.41)	–0.03 (−0.12–0.14)
PFOS	47	0.63 (0.43–0.86)	0.54	1.2 (0.97–1.4)	0.44 (0.39–0.53)	0.73 (0.44–1.0)

a Ranges are based on low- and high-bound estimates. Abbreviations: perfluoroheptanoic acid (PFHpA); perfluorononanoic acid (PFNA); perfluoroundecanoic acid (PFUnDA). n.d. = not detected.

### PFAA Input to Lake Vättern

A net input of at least 0.1 kg/year was calculated for all PFAAs except for PFHxS. Most surface water samples contained PFHxS in the same concentration range (0.56 ± 0.96 ng/L) as the inventory of Lake Vättern (0.69 ± 0.08 ng/L), and the zero net change is a result of the outflow being equal to the inflow (m^3^/year). Both atmospheric deposition and surface water inflow seem to be relevant sources for the input of PFCAs to Lake Vättern, with atmospheric deposition accounting for approximately 48% (PFOA) to 84% (PFDA) of the total input of the PFAAs included in the mass balance based on median estimates of input via surface water inflow. The largest input was observed for TFA and PFBA followed by PFPrA and PFOA, resulting in increases of 150, 4, 1.6, and 1.5 kg/year, respectively.

### PFAA Input from Atmospheric Deposition

The input from atmospheric deposition accounted for at least 48% of the total input for all PFCAs but for less than 20% of the total input of TFMS, PFBS, and PFHxS. For PFOS, atmospheric deposition seems to be a more important source, contributing to 45% to the total input. The contribution of atmospheric deposition to the total input of PFHxA (35–87%), PFOA (43–56%), PFDA (64–94%), and PFOS (39–56%) to Lake Vättern was somewhat higher than what has been observed in the Baltic Sea based on concentrations in atmospheric deposition samples collected in 2007–2008 (PFHxA, 11–37%; PFOA, 34–43%; PFDA, 31–72%; and PFOS, 20–21%).^5^

Considering the catchment area of 4500 km^2^ (6400 km^2^ including the lake surface), atmospheric deposition could be an important source of PFCAs to Lake Vättern via surface water inflow. Based on the PFCA concentration in atmospheric deposition and the amount of precipitation over the time of the study, the catchment area of Lake Vättern, excluding the lake surface, would receive an input of 230 kg/year (TFA), 3.5 kg/year (PFPrA), 7.9 kg/year (PFBA), and 3.5 kg/year (PFOA). A certain amount is likely taken up or retained by plants and soil,^41−43^ but an unknown fraction, which is larger for the smaller and more mobile PFCAs like TFA, is most probably transported to the lake. This subject will be further discussed in a later section.

**Figure 1:**
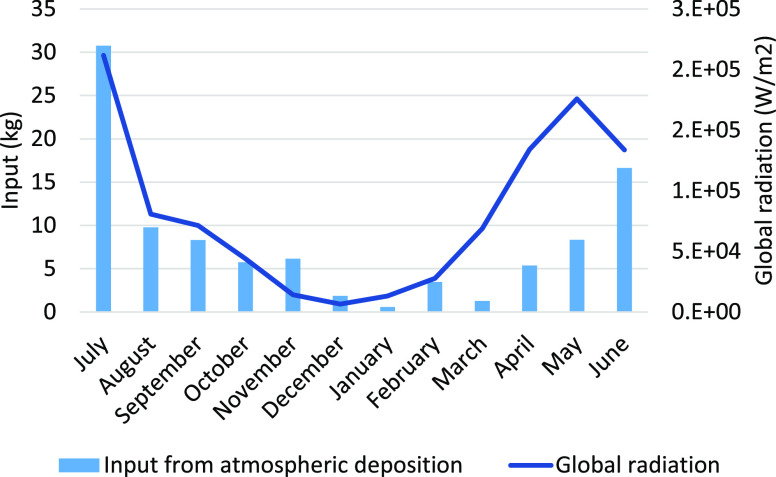
TFA input from atmospheric deposition (kg) and global radiation (W/m^2^) per month from July 2018 to June 2019.

The highest input via atmospheric deposition was observed for TFA, which is a well-known degradation product of HFCs and HCFCs.^7^ A higher input was observed during the summer months compared to the winter months, and a clear relationship between the TFA input via atmospheric deposition and global radiation was observed (Figure 1). This finding is in accordance with previous observations, and the measured concentrations are in the same range as reported in atmospheric deposition in Germany.^28^ A similar trend with higher input from atmospheric deposition during the summer months was also seen for most PFCAs except for PFPeA and PFOA (Figure S3). To assess the importance of atmospheric degradation of precursor compounds to the lake, the relationship between atmospheric hydroxyl radical concentrations and precursor degradation products (in this instance, PFCA input via atmospheric deposition) was investigated. Since hydroxyl radicals are produced by incoming global radiation into the atmosphere,^44^ the global radiation (W/m^2^), retrieved from measurements by the Swedish Meteorological and Hydrological Institute, was used to access the seasonal variations in hydroxyl radicals in the atmosphere over Lake Vättern. Significant positive monotonic correlation was observed between the global radiation and the input of TFA (ρ = 0.7, *P* < 0.01), PFBA (ρ = 0.7, *P* < 0.05), PFHxA (ρ = 0.7, *P* < 0.05), and PFUnDA (ρ = 0.6, *P* < 0.05). This finding suggests that hydroxyl radical-driven atmospheric oxidation of volatile precursors is an important source of PFCAs to the lake via atmospheric deposition except for PFPeA and PFOA, for which other sources to the atmosphere might also play an important role.^45^

The flux of PFCAs was low relative to the global radiation during March–May compared to June–August. This may be linked to a lower atmospheric concentration of volatile precursors during March–May, which can be influenced by factors such as temperature and emission rates. Positive monotonic correlation was observed between the flux of all PFCAs and temperature (ρ = 0.7, *P* < 0.01) except for PFOA. Seasonal trends of volatile PFASs and a relationship with temperature have previously been documented.^46^ In addition, seasonal differences in emission rates of volatile precursors used in automobile and domestic air conditioning can result in larger local and seasonal differences in TFA flux^47^ as a result of a shift from HFC-134a to HFO-1234yf, as the atmospheric lifetime with respect to oxidation by hydroxyl radicals is considerably shorter for HFO-1234yf (∼11 days)^48^ compared to HFC-134a (∼14 years).^49^

A higher input of PFOS via atmospheric deposition was observed in July 2018 when the global radiation was high; however, during the rest of the sampling campaign, the input via atmospheric deposition was not related to global radiation. No relationship was observed between input via atmospheric deposition and global radiation for TFMS, PFBS, or PFHxS.

### PFAA Input from Surface Water Inflow

**Figure 2:**
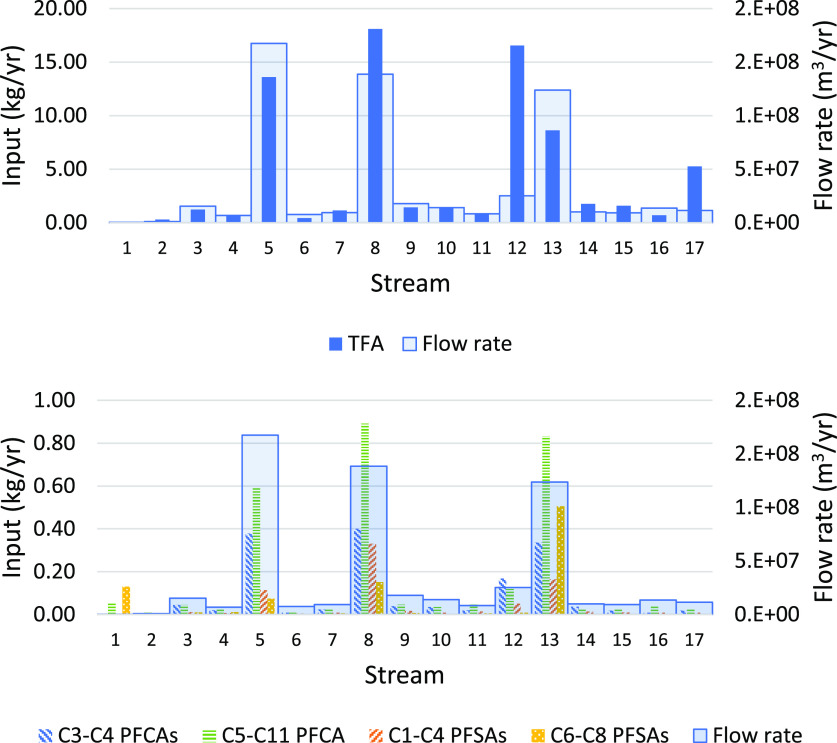
PFAA input from surface water inflow (kg/year) and the annual flow (m^3^/year).

Three streams were found to be major sources of PFAAs. These account for 74% of the total water inflow and for 55% (TFA) to 90% (PFHpA) of the PFAA input via surface water inflow based on median estimates (11–72% of the total input). In general, higher input of both PFCAs and PFSAs was observed from streams with a higher flow rate with some exceptions (Figure 2). In some streams, the concentration was dependent on the flow rate, and an increased flow rate seems to result in either lower (dilution) or higher concentration. Higher concentration was observed with increased flow rate in a stream receiving stormwater from a landfill and could possibly be explained by increased leaching because of heavy rainfall.

Two streams (streams 1 and 13) were found to be important sources of PFPeA, PFHxS, and PFOS to Lake Vättern. Both streams either directly or indirectly receive stormwater from areas with known previous use of AFFF. Stream 1 contributes with less than 0.1% of the water inflow to the lake but account for 12, 16, and 13% of the input of PFPeA, PFHxS, and PFOS, respectively, via surface water inflow (4–13% of the total input). Stream 13 contributes with 21% of the water inflow and account for 68, 47, and 58% of the input of PFPeA, PFHxS, and PFOS, respectively, via surface water inflow (23–39% of the total input). A third stream, which receives stormwater from a landfill, was identified as a source of PFPeA (stream 2), accounting for 2% of the input of PFPeA via surface water inflow (1% of the total input).

Five streams were identified as major sources of TFA (Figure 2). Three of these are the same streams that were identified as major input sources of most PFAAs and together account for 55% of the TFA input via surface water inflow (24% of the total TFA input). The high input of TFA via streams 5, 8, and 13 is likely a result of a large catchment area (840, 660, and 240 km^2^, respectively) receiving a large amount of atmospheric deposition resulting in an input of TFA of 88 kg/year. A positive monotonic correlation was observed between TFA input (kg/year) and catchment area (km^3^) (ρ = 0.9, *P* < 0.01). The other two main sources of TFA (streams 12 and 17) only account for 4 and 2% of the surface water inflow, respectively, but account for 22 and 7% of the TFA input via surface water inflow (10 and 3% of the total TFA input). Stream 12 has a relatively large catchment area (420 km^2^), while the catchment area of stream 17 is relatively low (84 km^2^). These two streams are not subject to any known contamination sources but are surrounded by agricultural activity. TFA has been identified as a main metabolite of pesticides containing a trifluoromethyl structure.^50,51^ Agriculture could therefore potentially be an important source of TFA contamination, but this could not be verified in the present project, and from the present data, atmospheric deposition seems to be the dominant route for TFA contamination.

Both surface water inflow and atmospheric deposition were found to be important sources of PFAAs to Lake Vättern. Atmospheric deposition seemed to be the main pathway for input of most PFCAs, including TFA. The input of TFA is also hypothesized to originate from agriculture, although the role of fluorine-containing pesticides as a source of contamination of freshwater lakes needs further investigation. From the catchment area of Lake Vättern, it is also possible that atmospheric deposition could account for PFAA input via surface water inflow. Positive monotonic correlation was observed between the catchment area (km^3^) and the input (kg/year) of all PFCAs (ρ > 0.7, *P* < 0.01) except PFPeA, PFHxA, and PFHpA. Among the PFSAs, only TFMS input was correlated with the catchment area (ρ > 0.9, *P* < 0.01). The correlation between the input and the catchment area might also be associated with a larger number of local sources such as sewage treatment plants.

The observed correlations between global radiation and the PFCA input indicate that atmospheric oxidation of volatile precursors is a major source of C_2_–C_11_ PFCAs to the lake except for PFPeA and PFOA, which are also influenced by other local anthropogenic sources. In precipitation samples from the Great Lakes, concentrations of PFBA were comparable across locations and years (2006–2018), while PFOS, PFOA, PFNA and PFDA concentrations decreased over time, which is suggested as a result of phaseouts and regulatory measures.^32^ PFBA has previously been suggested to be uniformly distributed in the atmosphere.^52^ Here, we suggest future monitoring of TFA and PFPrA to confirm that ultrashort-chain PFAAs are also distributed in the global atmosphere. Local anthropogenic sources seem to have a higher influence on most PFSAs, including ultrashort-chain PFSAs.

The main output was assumed to be via the main outflow. Other output pathways such as transformation, chemical and/or photochemical degradation in the water column, sedimentation, and volatilization were considered of minor importance and not included in the study. Phototransformation in the water column was found to only account for 0.03–0.4% of the total output of PFHxA, PFOA, PFDA, and PFOS in the Baltic Sea.^5^ Other transformation mechanisms in water are not known. Sedimentation as well as uptake in biota could be an important sink for long-chain PFAAs^5^ but are not considered relevant for ultrashort-chain PFAAs due to the high polarity of these compounds. The relevance of volatilization or potential transfer to the atmosphere via evaporation or formation of aerosols from the breaking of waves is not known.

The estimated input of several of the measured PFAAs to Lake Vättern exceeds the output. This positive net change will result in increased PFAA concentration in the water column over time. The doubling time for the PFAA concentration in the lake can be estimated by comparing the net change with the inventory in the lake, which is relevant for TFA (15–22 years), PFPRA (19–29 years), and PFBA (9–17 years) based on low- and high-bound estimates. Accumulation of persistent anthropogenic compounds in drinking water sources is a cause of concern solely based on the persistence criteria,^53^ especially for these highly polar compounds requiring combinations of advanced water treatment systems for their removal.^54^

The current study has assessed the relevance of atmospheric deposition and surface water inflow to PFAA contamination in a large drinking water source in Sweden over a 1 year time period, including input from 19 streams and atmospheric deposition at one site, with the assumption that the composition and amount of deposition is equal over the entire lake. Precipitation samples across the Great Lakes show that short-chain PFAA are evenly distributed in the global atmosphere, while concentrations of PFOS and PFOA are greater in urbanized/industrialized areas as compared to more remote locations.^32^ A larger set of samples would, in addition to less uncertainty in the reported concentration ranges, allow for detection of seasonal variations in PFAA input via inflowing streams that could possibly be attributed to agricultural activities and/or seasonal variations in vegetation. Since ultrashort-chain PFAAs can be taken up by plants,^18,19^ the input via inflowing streams could follow the life cycle of vegetation, with larger input being associated with decomposition during fall.

The positive net input of PFAAs to Lake Vättern warrants further monitoring of these compounds. Precipitation and surface water is shown here as suitable matrices for monitoring of short- and ultrashort-chain PFAA, providing additional data currently scarce in the scientific literature.

## Supporting Information Available

Detailed information about sampling locations and contamination situation, catchment area and annual flow of inflowing surface water; properties of Lake Vättern; sample preparation and sample analysis; list of target analytes; abbreviations; MRM transitions; separation method and internal standards used for quantification; limits of detection; repeatability; extraction efficiencies; concentrations of target analytes; figures illustrating the mass balance and correlation between PFAA input from atmospheric deposition with global radiation (PDF)
