# Supplementary material for: Mass Balance of Perfluoroalkyl Acids, Including Trifluoroacetic Acid, in a Freshwater Lake
Source: Environ Sci Technol. 2021 Dec 20;56(1):251–9. doi: 10.1021/acs.est.1c04472 (PMC8733927; doi:10.1021/acs.est.1c04472)
Supplement: Supplementary file 1 — es1c04472_si_001.pdf [file es1c04472_si_001.pdf]

Supporting Information

Mass balance of perfluoroalkyl acids including trifluoroacetic acid in a freshwater lake

Maria K. Björnsdotter,[a]* Leo W. Y. Yeung,[a] Anna Kärrman,[a] Ingrid Ericson Jogsten[a]

[a]Man-Technology-Environment Research Centre (MTM), Örebro University, 701 82 Örebro, Sweden.

*Corresponding author

E-mail: maria.bjornsdotter@oru.se

Number of tables: 15, number of figures: 3, number of pages: 22.

Table of Contents

Chemicals and Reagents	**S3**
**Figure S1.** Geographical location and sampling sites in and around Lake Vättern	**S4**
**Table S1.** Sampling locations, known potential contamination sources, catchment area and annual flow	**S5**
Sample Extraction	**S5**
Sample Analysis	**S6**
**Table S2.** Target analytes, abbreviation, MRM transitions, separation method and internal standard used for quantification	**S7**
**Table S3.** Limits of detection, recovery, and repeatability	**S8**
**Table S4.** Properties of Lake Vättern	**S8**
**Table S5.** PFAA concentration in atmospheric deposition, hours of sunlight per day, global radiation and amount of rain	**S9**
**Table S6.** Atmospheric deposition fluxes of PFAAs	**S10**
**Table S7.** PFAA concentration and flow rate in streams 1-3	**S11**
**Table S8.** PFAA concentration and flow rate in streams 4-6	**S12**
**Table S9.** PFAA concentration and flow rate in streams 7-9	**S13**
**Table S10.** PFAA concentration and flow rate in streams 10-12	**S14**
**Table S11.** PFAA concentration and flow rate in streams 13-15	**S15**
**Table S12.** PFAA concentration and flow rate in streams 16-18	**S16**
**Table S13.** PFAA concentration and flow rate in streams 19 (outflow) and 20 (reference)	**S17**
**Table S14.** PFAA concentration and flow rate in effluent from the sewage treatment plant and the paper mill	**S18**
**Table S15.** PFAA concentration in surface water in Lake Vättern	**S19**
**Figure S2.** Mass balance of PFAAs in Lake Vättern	**S20**
**Figure S3**. PFAA input from atmospheric deposition and global radiation	**S21**
**References**	**S22**

Chemicals and Reagents

Ten perfluoroalkyl carboxylic acids (PFCAs), including TFA, PFPrA, perfluorobutanoic acid (PFBA), perfluoropentanoic acid (PFPeA), perfluorohexanoic acid (PFHxA), perfluoroheptanoic acid (PFHpA), perfluorooctanoic acid (PFOA), perfluorononanoic acid (PFNA), perfluorodecanoic acid (PFDA), and perfluoroundecanoic acid (PFUnDA), and four perfluoroalkyl sulfonic acids (PFSAs), including TFMS, perfluorobutane sulfonic acid (PFBS), perfluorohexane sulfonic acid (PFHxS), and perfluorooctane sulfonic acid (PFOS), were measured. Standards of native PFCAs (C_4-C_{11}) and PFSAs (C_4-C_8) and mass-labelled standards of PFCAs (C_4-C_{11}), PFSAs (C_4-C_8) were from Wellington laboratories (Guelph, ON, Canada). TFA was purchased from Sigma-Aldrich, (Munich, Germany). PFPrA was from Sigma-Aldrich, (Oakville, ON, Canada). TFMS was from Sigma-Aldrich, (Stockholm, Sweden). Mass-labelled standard of TFA ($^{13}C_2$-TFA) was purchased from Toronto Research Chemicals Inc, (Toronto, ON, Canada). Mass-labelled standards for PFPrA and TFMS were not available. The purity of all standards was above 97%. Glass microfiber filters (Whatman), ammonium acetate, and glacial acetic acid were purchased from Sigma-Aldrich, (Stockholm, Sweden). Ammonia solution (NH_4OH) and methanol (MeOH) were from Fischer Scientific, (Ottawa, ON, Canada). Weak anion exchange solid-phase extraction (WAX-SPE) cartridges were obtained from Waters Corporation, (Milford, MA, USA).

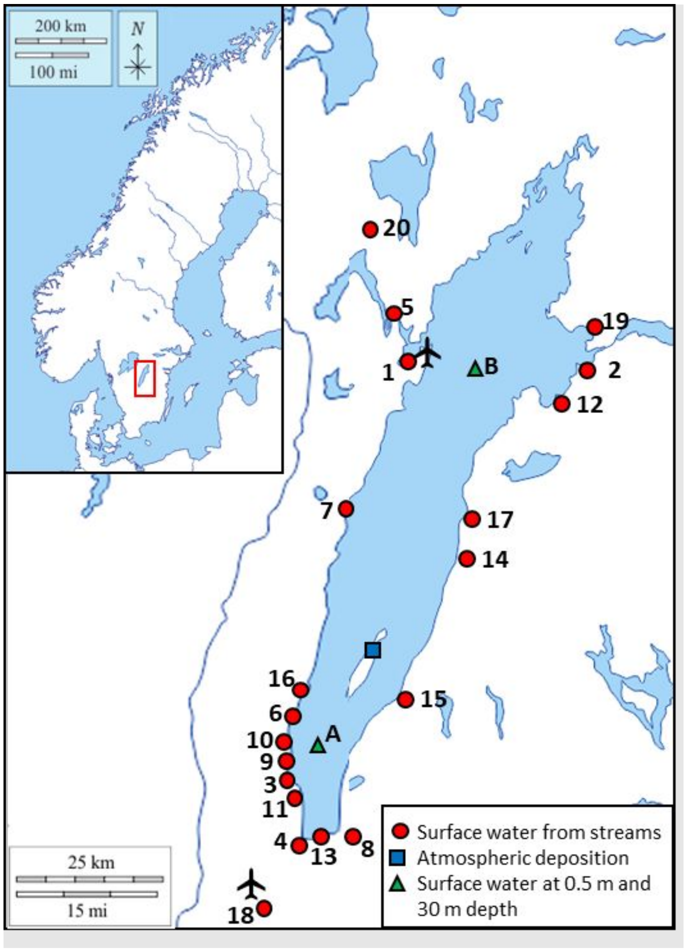

Figure S1. Geographical location and sampling sites in and around Lake Vättern. Maps are based on https://d-maps.com/.

Table S1. Sampling locations, known potential contamination sources, catchment area (km^2) and annual flow (m^3/yr). Sites with known use of aqueous film-forming foam (AFFF) are marked as AFFF. Fire-fighting training sites (FFTSs) with no known use of AFFF are marked as FFTS. Other contamination refers to sites with known contamination with metals and/or organic pollutants other than those defined as PFASs.

Stream	Sampling location	Potential contamination sources	Catchment area (km^2)	Flow rate (m^3/yr)
1	Kärnebäcken	AFFF	NA	326 000
2	Hamrabäcken	Landfill	NA	932 000
3	Domneån	Other contamination	66	15 300 000
4	Dunkehallaån	AFFF, industry, stormwater	26	6 840 000
5	Forsviksån	Other contamination	840	167 000 000
6	Gagnån	STP, Industry	29	7 560 000
7	Hjoån	Other contamination, stormwater, individual sewers	63	9 340 000
8	Huskvarnaån	PFAS contamination, landfill, STP, stormwater	660	139 000 000
9	Hökesån	Firefighting training site, industry, stormwater	69	17 900 000
10	Knipån	Individual sewers	53	14 000 000
11	Lillån	STP, other contamination	35	8 400 000
12	Mjölnaån	Agriculture, individual sewers	420	25 200 000
13	Munksjön	AFFF, STP, stormwater, industry	240	124 000 000
14	Orrnäsaån	Agriculture	64	9 980 000
15	Röttleån	Individual sewers	220	9 180 000
16	Svedån	No known impact	49	13 500 000
17	Ålebäcken	Agriculture	84	11 500 000
18	Sandserydsån[a]	AFFF	NA	NA
19	Motala ström[b]	NA	6 400	576 000 000
20	Unden[c]	No known impact	NA	NA

Abbreviations: Aqueous film-forming foam (AFFF) (sites with known use of AFFF); fire-fighting training site (FFTS) (no known use of AFFF); Sewage treatment plant (STP); Not available (NA).

[a]Not included in mass balance. The stream discharges into a small lake, which is connected to Lake Vättern via stream number 13.

[b]Surface water outflow.

[c]Reference sample collected upstream the study area.

Sample Extraction

The SPE cartridges were preconditioned with 4 mL 0.1% NH_4OH in MeOH followed by 4 mL MeOH and 4 mL ultra-pure water. The samples were then loaded at approximately 1 drop/s through the cartridges. After loading the samples, the cartridges were washed by passage of 4 mL ultra-pure water followed by 4 mL ammonium acetate buffer solution (pH 4) and then dried under vacuum for 30 min before elution of target analytes. The neutral fraction was eluted by adding 4 mL MeOH. The anionic

fraction was then eluted by adding 4 mL 0.1% NH_4OH in MeOH. In the present study, only the anionic fraction was analyzed. The eluate was evaporated to approximately 0.5 mL at 60 °C and 400 mbar, transferred to an LC vial and then further evaporated to 250 µL under a gentle stream of nitrogen. Mass-labelled internal standards and recovery standards were added prior to extraction and prior to injection, respectively, to monitor the recovery of the method. For quantification of C1-C4 PFAAs, 50 µL of the extract was transferred to a new LC vial and 75 µL MeOH was added. Aliquots of 2 µL were injected into the supercritical fluid chromatography (SFC) tandem mass spectrometry system (MS/MS). For quantification of C5-C12 PFAAs, 50 µL was transferred to new a LC vial and 75 µL of 2 mM aqueous ammonium acetate was added. Aliquots of 10 µL were injected into the ultra performance liquid chromatography (UPLC)-MS/MS system.

Sample Analysis

The mobile phase for SFC-MS/MS analysis consisted of CO_2 (A) and 0.1% NH_4OH in MeOH (B). The gradient of the mobile phase started with an initial B concentration of 15%, which was then increased to 35% over 5 min. This was held for 1 min before returning to initial conditions over 1 min. The flow rate was 1.2 mL/min and the active back pressure regulator was set at 2 000 psi throughout the chromatographic separation. To prevent instrumental contamination, an isolator column (PFC Isolator) (Waters Corporation, Milford, MA, USA) was inserted between the pump and the injector. The mobile phase for UPLC-MS/MS analysis consisted of 2 mM NH_4Ac in MeOH:H_2O (3:7) (A) and 2 mM NH_4Ac in MeOH (B) at a flow rate of 0.3 mL/min. The gradient of the mobile phase started with an initial B concentration of 1% for 1 min. This was then increased to 100% over 12 min and held for 1 min and finally re-conditioned for 3 min. The source parameters (for both instrumental setups) were set as following: capillary voltage, 0.7 kV; source temperature, 150 °C; desolvation temperature, 400 °C; cone gas flow, 150 L/h; desolvation gas flow, 800 L/h; collision gas flow, 0.2 mL/min; nebulizer, 6.5 bar.

Table S2. Target analytes, abbreviation, MRM transitions, separation method and internal standard used for quantification.

Target analyte	Abbreviation	1st MRM transition	2nd MRM transition	Separation method	Internal standard
Trifluoroacetic acid	TFA	112.90 > 68.96	-	SFC	^{13}C-M2TFA
Perfluoropropanoic acid	PFPrA	162.97 > 118.90	-	SFC	^{13}C-M4PFBA
Perfluorobutanoic acid	PFBA	212.97 > 169.00	-	SFC	^{13}C-M4PFBA
Perfluoropentanoic acid	PFPeA	262.97 > 219.00	-	UPLC	^{13}C-M3PFPeA
Perfluorohexanoic acid	PFHxA	312.97 > 269.00	312.97 > 118.95	UPLC	^{13}C-M2PFHxA
Perfluoroheptanoic acid	PFHpA	362.97 > 319.00	362.97 > 168.97	UPLC	^{13}C-M4PFHpA
Perfluorooctanoic acid	PFOA	412.97 > 369.00	412.97 > 168.97	UPLC	^{13}C-M4PFOA
Perfluorononanoic acid	PFNA	462.99 > 419.00	462.99 > 219.00	UPLC	^{13}C-M5PFNA
Perfluorodecanoic acid	PFDA	512.97 > 469.00	512.97 > 219.00	UPLC	^{13}C-M2PFDA
Perfluoroundecanoic acid	PFUnDA	562.97 > 268.99	562.97 > 519.00	UPLC	^{13}C-M2PFUnDA
Trifluoromethane sulfonic acid	TFMS	149.12 > 79.91	149.12 > 98.95	SFC	^{13}C-M3PFBS
Perfluorobutane sulfonic acid	PFBS	298.90 > 79.96	298.90 > 98.90	SFC	^{13}C-M3PFBS
Perfluorohexane sulfonic acid	PFHxS	398.90 > 79.96	398.90 > 98.90	UPLC	^{18}O-M2PFHxS
			398.90 > 119.01		
			398.90 > 319.00		
Perfluorooctane sulfonic acid	PFOS	498.97 > 79.96	498.97 > 98.96	UPLC	^{13}C-M4PFOS
			498.97 > 169.03		
			498.97 > 419.00		

Table S3. Limits of detection (LOD) (ng/L), recovery (%) and repeatability (%) based on spiked blank samples (ultra-pure water) and spiked test samples (surface water) for monitoring av PFAAs in water samples connected to Lake Vättern.

	LOD (ng/L)				
	**Atmospheric deposition (*n*=8)**	**Surface water and effluent (*n*=8)**	**Lake water (*n*=8)**	**Recovery ± SD (%) (*n*=11)**	**Repeatability (%) (*n*=11)**
**TFA**	0.10	0.10	0.05	80 ± 22	11%
**PFPrA**	0.10	0.60	0.30	72 ± 12	15%
**PFBA**	1.50	0.10	0.05	94 ± 29	16%
**PFPeA**	0.03	0.07	0.04	84 ± 23	15%
**PFHxA**	0.03	0.25	0.12	85 ± 20	17%
**PFHpA**	0.03	0.03	0.01	86 ± 18	14%
**PFOA**	0.16	0.31	0.15	83 ± 18	14%
**PFNA**	0.03	0.03	0.01	86 ± 20	17%
**PFDA**	0.03	0.04	0.02	86 ± 20	13%
**PFUnDA**	0.03	0.10	0.05	87 ± 24	14%
**TFMS**	0.10	0.10	0.05	87 ± 21	12%
**PFBS**	0.10	0.10	0.05	90 ± 18	17%
**PFHxS**	0.03	0.03	0.01	89 ± 14	16%
**PFOS**	0.03	0.15	0.08	88 ± 16	17%

Table S4. Properties of Lake Vättern. Data were retrieved from the Swedish Meteorological and Hydrological Institute[1] and by oral communication with the water management association Vätternvårdsförbundet.

Surface area of Lake Vättern (km^2)	1885
Catchment area (km^2)	6376
Volume (km^3)	74
Average depth (m)	40
Maximum depth (m)	120
Residence time (years)	60–70
Average water flow (m^3/s)	40*
Precipitation (catchment area) (mm/year)	650
Evaporation (catchment area) (mm/year)	450
Runoff (mm/year)	200

*The average water flow is based on measured and modelled data during 1940–2020. The average water flow has been lower since 2016 and was 18 m^3/s during the year of the study (2019).

Table S5. PFAA concentration (ng/L) in atmospheric deposition during the sampling period from July 2018 to June 2019.

Sampling month	July	August	September	October	November	December	January	February	March	April	May	June
TFA	230	81	100	48	67	24	18	47	29	300	160	150
PFPrA	3.7	1.4	1.2	1.1	1.2	0.92	0.94	1.2	0.93	1.9	1.4	1.3
PFBA	6.7	3.5	6.4	<1.5	<1.5	<1.5	<1.5	6.1	4.4	6.2	4.3	5.0
PFPeA	0.25	0.25	0.21	0.22	0.38	0.16	0.19	0.32	0.28	0.70	0.52	0.31
PFHxA	1.0	0.42	0.52	0.33	0.43	0.20	0.27	0.55	0.42	2.2	0.79	0.54
PFHpA	0.71	0.44	0.57	0.33	0.52	0.28	0.29	0.36	0.42	1.3	0.69	0.85
PFOA	1.5	0.60	1.2	1.7	1.7	1.5	1.7	1.7	1.5	2.9	1.9	2.0
PFNA	0.84	0.16	0.35	0.28	0.20	0.11	0.17	0.17	0.13	0.52	0.33	0.38
PFDA	0.49	0.09	0.13	0.13	0.13	0.06	0.09	0.08	0.09	0.24	0.12	0.16
PFUA	0.28	0.06	0.06	0.08	0.07	0.03	0.05	0.04	0.04	0.17	0.12	0.10
PFDoDA	0.11	<0.03	<0.03	<0.03	0.06	<0.03	<0.03	<0.03	<0.03	<0.03	0.05	<0.03
TFMS	0.15	<0.10	<0.10	<0.10	0.13	<0.10	<0.10	<0.10	<0.10	<0.10	<0.10	<0.10
PFEtS	<0.10	<0.10	<0.10	<0.10	<0.10	<0.10	<0.10	<0.10	<0.10	<0.10	<0.10	<0.10
PFPrS	<0.10	<0.10	<0.10	<0.10	<0.10	<0.10	<0.10	<0.10	<0.10	<0.10	<0.10	<0.10
PFBS	<0.03	0.09	0.06	0.10	0.07	0.10	0.08	0.09	0.05	0.08	0.03	0.05
PFPeS	<0.03	<0.03	<0.03	<0.03	<0.03	<0.03	<0.03	<0.03	<0.03	<0.03	<0.03	<0.03
PFHxS	<0.03	0.04	0.14	0.10	0.06	0.06	0.09	0.12	0.06	0.14	0.06	<0.03
PFHpS	<0.1	<0.1	<0.1	<0.1	<0.1	<0.1	<0.1	<0.1	<0.1	<0.1	<0.1	<0.1
PFOS	2.1	0.12	0.58	0.45	0.23	0.24	0.36	0.30	0.25	0.61	0.21	0.35
PFNS	<0.1	<0.1	<0.1	<0.1	<0.1	<0.1	<0.1	<0.1	<0.1	<0.1	<0.1	<0.1
PFDS	<0.03	<0.03	<0.03	<0.03	<0.03	<0.03	<0.03	<0.03	<0.03	<0.03	<0.03	<0.03
PFDoDS	<0.03	<0.03	<0.03	<0.03	<0.03	<0.03	<0.03	<0.03	<0.03	<0.03	<0.03	<0.03

Table S6. Atmospheric deposition fluxes of PFAAs (ng/m^2), hours of sunlight per day (h), global radiation (W/m^2), and amount of rain (mm) during the sampling period from July 2018 to June 2019. Fluxes in the case of concentrations below the LOD is calculated based on the LOD for each PFAA and the amount of precipitation for each month. The meteorological parameters are based on measurements by the Swedish Meteorological and Hydrological Institute at a meteorological station on the same island as the precipitation samples were collected (amount of rain) or at four stations within a radius of 130–160 km from the sampling location (average data) (hours of sunlight per day and global radiation).

Sampling month	July	August	September	October	November	December	January	February	March	April	May	June
Hours of sunligt per day (h)	370	126	130	128	48	29	50	84	130	283	259	206
Global radiation (W/m^2)	212000	81000	71000	44000	14000	7000	13000	28000	69000	134000	176000	134000
Amount of rain (mm)	69	64	42	63	48	42	17	39	23	9	27	59
TFA	16 000	5 100	4 400	3 000	3 200	990	300	1800	670	2 800	4 400	8 800
PFPrA	260	87	51	69	58	38	16	46	22	18	36	76
PFBA	460	220	270	<92	<71	<61	<24	240	100	58	120	290
PFPeA	18	16	8.6	14	18	6.5	3.1	13	6.6	6.6	14	18
PFHxA	71	27	22	21	21	8.4	4.5	21	10	20	21	32
PFHpA	50	28	24	21	25	12	4.9	14	10	12	18	50
PFOA	110	38	49	110	82	63	28	66	35	27	49	120
PFNA	58	10	15	18	10	4.7	2.8	6.7	3.1	4.9	8.9	22
PFDA	34	5.7	5.3	8.3	6.1	2.5	1.5	3.1	2.2	2.3	3.2	9.2
PFUA	20	4.0	2.7	4.9	3.3	1.3	0.9	1.6	1.0	1.6	3.2	5.8
PFDoDA	7.8	<1.6	<1.0	<1.6	2.9	<1.0	<0.41	<1.0	<0.58	<0.24	1.2	<1.5
TFMS	10	<6.4	<4.2	<6.3	6.5	<4.2	<1.7	<3.9	<2.3	<0.94	<2.7	<5.9
PFEtS	<6.9	<6.4	<4.2	<6.3	<4.8	<4.2	<1.7	<3.9	<2.3	<0.94	<2.7	<5.9
PFPrS	<6.9	<6.4	<4.2	<6.3	<4.8	<4.2	<1.7	<3.9	<2.3	<0.94	<2.7	<5.9
PFBS	<1.7	5.7	2.3	6.0	3.6	4.1	1.3	3.7	1.2	0.7	0.9	2.9
PFPeS	<1.7	<1.6	<1.0	<1.6	<1.2	<1.0	<0.41	<1.0	<0.58	<0.24	<0.67	<1.5
PFHxS	<1.7	2.5	5.8	6.1	3.1	2.6	1.4	4.9	1.3	1.3	1.7	<1.5
PFHpS	<6.9	<6.4	<4.2	<6.3	<4.8	<4.2	<1.7	<3.9	<2.3	<0.94	<2.7	<5.9
PFOS	150	7.8	24	28	11	10	6.0	12	5.9	5.8	5.6	20
PFNS	<6.9	<6.4	<4.2	<6.3	<4.8	<4.2	<1.7	<3.9	<2.3	<0.94	<2.7	<5.9
PFDS	<1.7	<1.6	<1.0	<1.6	<1.2	<1.0	<0.41	<1.0	<0.58	<0.24	<0.67	<1.5
PFDoDS	<1.7	<1.6	<1.0	<1.6	<1.2	<1.0	<0.41	<1.0	<0.58	<0.24	<0.67	<1.5

Table S7. PFAA concentration (ng/L) and flow rate (m^3/s) in streams 1-3 during the sample collection from March to December 2019.

Stream	1				2				3			
Sample location	Kärnebäcken				Hamrabäcken				Domneån			
Sampling month	March	June	September	December	April	June	September	December	March	June	September	December
Flow rate m^3/s	0.012	0.006	0.003	0.121	0.026	0.006	0.001	0.255	0.816	0.169	0.240	1.220
TFA	160	130	170	230	410	230	69	630	68	NA	93	120
PFPrA	2.8	2.8	2.8	2.9	64	1.6	0.66	0.63	0.65	NA	1.2	0.75
PFBA	7.2	6.3	9.2	12	<0.10	6.4	1.4	1.5	2.30	NA	2.0	2.8
PFPeA	56	41	49	51	1.6	32	4.2	<0.07	<0.07	NA	5.4	<0.07
PFHxA	75	43	47	61	1.4	30	2.1	1.7	0.49	NA	0.75	<0.25
PFHpA	28	23	25	27	0.84	20	1.7	1.9	0.44	NA	0.73	<0.03
PFOA	31	24	22	24	2.6	31	3.5	3.7	1.6	NA	3.4	2.8
PFNA	1.6	4.7	3.1	1.6	<0.03	0.87	<0.03	<0.03	<0.03	NA	0.28	<0.03
PFDA	0.18	0.58	0.62	<0.04	<0.04	0.30	<0.04	<0.04	<0.04	NA	<0.04	<0.04
PFUnDA	<0.10	0.22	0.18	<0.10	<0.10	<0.10	<0.10	<0.10	<0.10	NA	<0.10	<0.10
PFDoDA	<0.03	<0.03	<0.03	<0.03	<0.03	<0.03	<0.03	<0.03	<0.03	NA	<0.03	<0.03
TFMS	0.81	0.88	0.65	0.87	0.99	15	0.40	0.89	0.35	NA	0.40	0.62
PFEtS	0.52	0.36	<0.24	0.43	<0.24	0.39	<0.24	0.25	<0.24	NA	<0.24	<0.24
PFPrS	3.5	1.90	1.2	2.8	4.0	1.6	<0.10	<0.10	<0.10	NA	<0.10	0.44
PFBS	22	8.80	7.5	14	0.41	7.1	0.29	0.51	0.28	NA	0.37	0.40
PFPeS	31	10	8.6	17	<0.03	2.1	<0.03	<0.03	0.07	NA	<0.03	<0.03
PFHxS	240	120	120	170	0.20	7.1	0.17	0.18	0.40	NA	0.52	<0.03
PFHpS	14	7.7	5.5	10	<0.10	0.44	<0.10	<0.10	<0.10	NA	<0.10	<0.10
PFOS	300	250	150	260	0.21	3.5	0.38	0.23	0.57	NA	0.65	0.35
PFNS	0.11	<0.10	<0.10	<0.10	<0.10	<0.10	<0.10	<0.10	<0.10	NA	<0.10	<0.10
PFDS	<0.03	<0.03	<0.03	<0.03	<0.03	<0.03	<0.03	<0.03	<0.03	NA	<0.03	<0.03
PFDoDS	<0.03	<0.03	<0.03	<0.03	<0.03	<0.03	<0.03	<0.03	<0.03	NA	<0.03	<0.03

Table S8. PFAA concentration (ng/L) and flow rate (m^3/s) in streams 4-6 during the sample collection from March to December 2019.

Stream	4				5				6			
Sample location	Dunkehallaån				Forsviksån				Gagnån			
Sampling month	March	June	September	December	March	June	September	December	March	June	September	December
Flow rate m^3/s	0.556	0.070	0.070	0.588	10.600	3.820	1.860	13.400	0.566	0.163	0.104	0.495
TFA	63	83	110	104	<0.10	83	83	80	54	43	58	71
PFPrA	0.65	1.3	0.99	0.77	<0.60	1.0	0.88	0.70	<0.60	<0.60	0.61	<0.60
PFBA	1.6	1.5	3.2	2.1	<0.10	1.3	1.6	1.8	0.47	0.69	1.1	1.9
PFPeA	<0.07	1.8	<0.07	<0.07	<0.07	<0.07	<0.07	<0.07	<0.07	<0.07	4.6	<0.07
PFHxA	0.85	1.1	<0.25	<0.25	0.58	0.38	0.48	<0.25	<0.25	<0.25	<0.25	<0.25
PFHpA	<0.03	0.75	<0.03	<0.03	<0.03	0.46	0.66	0.63	<0.03	0.21	<0.03	<0.03
PFOA	2.10	3.3	3.5	2.9	1.8	1.6	2.90	2.5	1.3	1.4	2.1	2.5
PFNA	<0.03	0.21	<0.03	0.48	0.25	0.37	0.33	0.23	<0.03	0.09	<0.03	<0.03
PFDA	<0.04	0.17	<0.04	<0.04	<0.04	0.09	0.12	<0.04	<0.04	<0.04	<0.04	<0.04
PFUnDA	<0.10	<0.10	<0.10	<0.10	<0.10	<0.10	<0.10	<0.10	<0.10	<0.10	<0.10	<0.10
PFDoDA	<0.03	<0.03	<0.03	<0.03	<0.03	<0.03	<0.03	<0.03	<0.03	<0.03	<0.03	<0.03
TFMS	0.30	0.40	0.40	0.31	1.7	0.41	0.54	<0.10	0.52	0.26	0.46	0.38
PFEtS	<0.24	0.25	<0.24	<0.24	<0.24	<0.24	<0.24	<0.24	0.32	0.26	<0.24	<0.24
PFPrS	0.45	<0.10	<0.10	0.44	<0.10	0.48	<0.10	<0.10	<0.10	<0.10	<0.10	0.45
PFBS	0.53	0.53	0.56	0.70	0.23	0.19	0.21	<0.10	0.25	<0.10	<0.10	0.32
PFPeS	<0.03	0.34	<0.03	<0.03	<0.03	<0.03	<0.03	<0.03	<0.03	<0.03	<0.03	<0.03
PFHxS	0.71	1.6	0.94	0.53	0.17	0.28	0.16	0.17	<0.03	0.08	0.10	0.20
PFHpS	<0.10	<0.10	<0.10	<0.10	<0.10	<0.10	<0.10	<0.10	<0.10	<0.10	<0.10	<0.10
PFOS	0.74	0.99	0.68	0.63	0.31	0.35	0.23	0.19	<0.15	<0.15	0.19	0.28
PFNS	<0.10	<0.10	<0.10	<0.10	<0.10	<0.10	<0.10	<0.10	<0.10	<0.10	<0.10	<0.10
PFDS	<0.03	<0.03	<0.03	<0.03	<0.03	<0.03	<0.03	<0.03	<0.03	<0.03	<0.03	<0.03
PFDoDS	<0.03	<0.03	<0.03	<0.03	<0.03	<0.03	<0.03	<0.03	<0.03	<0.03	<0.03	<0.03

Table S9. PFAA concentration (ng/L) and flow rate (m^3/s) in streams 7-9 during the sample collection from March to December 2019.

Stream	7				8				9			
Sample location	Hjoån				Huskvarnaån				Hökesån			
Sampling month	March	June	September	December	March	June	September	December	March	June	September	December
Flow rate m^3/s	0.638	0.355	0.110	0.639	13.600	4.000	0.100	14.500	1.400	0.305	0.188	1.250
TFA	<0.10	130	110	140	98	130	140	140	71	67	89	110
PFPrA	<0.60	1.1	1.4	0.67	0.71	2.4	1.1	0.78	<0.60	1.1	1.2	<0.60
PFBA	<0.10	1.5	1.8	1.9	1.5	2.3	3.4	1.7	1.1	1.0	1.8	2.2
PFPeA	<0.07	<0.07	<0.07	<0.07	<0.07	3.8	<0.07	<0.07	<0.07	<0.07	<0.07	<0.07
PFHxA	<0.25	0.36	<0.25	<0.25	1.1	2.6	1.9	<0.25	0.52	0.48	<0.25	<0.25
PFHpA	<0.03	0.53	0.49	<0.03	0.90	2.5	1.6	<0.03	0.34	0.34	<0.03	<0.03
PFOA	1.4	1.5	3.0	2.7	2.1	3.9	3.6	2.8	1.6	1.6	2.7	2.6
PFNA	<0.03	0.25	0.36	<0.03	0.24	0.50	0.52	<0.03	<0.03	0.13	<0.03	<0.03
PFDA	0.16	0.10	0.06	<0.04	<0.04	0.31	<0.04	<0.04	0.05	<0.04	<0.04	<0.04
PFUnDA	<0.10	<0.10	<0.10	<0.10	<0.10	0.12	<0.10	<0.10	<0.10	<0.10	<0.10	<0.10
PFDoDA	<0.03	<0.03	<0.03	<0.03	0.04	<0.03	<0.03	<0.03	<0.03	<0.03	<0.03	<0.03
TFMS	2.1	0.66	0.58	0.66	1.0	2.1	2.2	1.2	0.33	0.69	0.70	0.73
PFEtS	0.35	<0.24	0.25	<0.24	0.27	0.26	0.25	<0.24	<0.24	<0.24	<0.24	<0.24
PFPrS	<0.10	<0.10	<0.10	<0.10	0.45	0.50	0.51	<0.10	<0.10	<0.10	<0.10	<0.10
PFBS	0.32	0.20	0.23	0.39	0.47	1.2	0.95	0.49	0.27	0.19	0.24	0.46
PFPeS	<0.03	<0.03	<0.03	<0.03	<0.03	0.14	<0.03	<0.03	<0.03	<0.03	<0.03	<0.03
PFHxS	0.14	0.19	0.24	0.17	0.28	0.50	0.46	0.31	0.09	0.16	0.17	<0.03
PFHpS	<0.10	<0.10	<0.10	<0.10	<0.10	<0.10	<0.10	<0.10	<0.10	<0.10	<0.10	<0.10
PFOS	0.38	0.30	0.53	0.45	0.62	1.2	0.79	0.51	0.18	0.26	0.32	<0.15
PFNS	<0.10	<0.10	<0.10	<0.10	<0.10	<0.10	<0.10	<0.10	<0.10	<0.10	<0.10	<0.10
PFDS	<0.03	<0.03	<0.03	<0.03	<0.03	<0.03	<0.03	<0.03	<0.03	<0.03	<0.03	<0.03
PFDoDS	<0.03	<0.03	<0.03	<0.03	<0.03	<0.03	<0.03	<0.03	<0.03	<0.03	<0.03	<0.03

Table S10. PFAA concentration (ng/L) and flow rate (m^3/s) in streams 10-12 during the sample collection from March to December 2019.

Stream	10				11				12			
Sample location	Knipån				Lillån				Mjölnaån			
Sampling month	March	June	September	December	March	June	September	December	March	June	September	December
Flow rate m^3/s	1.350	0.150	0.083	1.040	0.726	0.051	0.035	1.000	0.341	0.153	0.081	2.340
TFA	79	90	110	110	<0.10	89	110	160	710	670	650	600
PFPrA	<0.60	1.1	0.81	0.60	<0.60	2.0	0.88	0.63	0.88	2.3	1.7	1.3
PFBA	1.4	1.4	2.1	2.8	<0.10	1.5	1.6	1.5	2.1	4.5	6.2	5.8
PFPeA	<0.07	<0.07	<0.07	<0.07	<0.07	2.0	2.7	<0.07	<0.07	<0.07	<0.07	<0.07
PFHxA	0.33	<0.25	<0.25	<0.25	<0.25	2.0	2.0	<0.25	0.48	0.96	1.1	<0.25
PFHpA	<0.03	<0.03	<0.03	<0.03	<0.03	1.0	1.3	0.48	0.42	1.1	1.4	0.84
PFOA	1.5	2.0	2.8	2.5	2.4	2.2	3.1	2.7	2.2	2.3	3.5	3.3
PFNA	0.13	<0.03	<0.03	<0.03	<0.03	2.2	0.38	<0.03	<0.03	0.53	0.47	<0.03
PFDA	0.06	<0.04	<0.04	<0.04	<0.04	0.17	<0.04	<0.04	<0.04	<0.04	<0.04	<0.04
PFUnDA	<0.10	<0.10	<0.10	<0.10	<0.10	<0.10	<0.10	<0.10	<0.10	<0.10	<0.10	<0.10
PFDoDA	<0.03	<0.03	<0.03	<0.03	<0.03	<0.03	<0.03	<0.03	<0.03	<0.03	<0.03	<0.03
TFMS	0.32	0.49	0.36	0.43	1.4	1.1	0.60	0.45	1.2	2.0	2.1	1.3
PFEtS	0.24	0.25	0.25	<0.24	<0.24	0.29	<0.24	<0.24	0.40	0.28	0.30	0.28
PFPrS	<0.10	<0.10	<0.10	0.44	<0.10	0.49	0.51	0.46	<0.10	<0.10	0.49	0.47
PFBS	0.23	0.21	0.20	0.33	0.46	1.2	1.0	0.43	0.18	0.37	0.45	0.30
PFPeS	<0.03	<0.03	<0.03	<0.03	<0.03	0.10	<0.03	<0.03	<0.03	<0.03	<0.03	<0.03
PFHxS	<0.03	<0.03	0.08	<0.03	0.30	0.38	0.28	0.22	<0.03	0.19	0.21	<0.03
PFHpS	<0.10	<0.10	<0.10	<0.10	<0.10	<0.10	<0.10	<0.10	<0.10	<0.10	<0.10	<0.10
PFOS	0.16	0.19	<0.15	0.41	0.34	0.71	0.46	0.29	0.22	0.20	0.33	<0.15
PFNS	<0.10	<0.10	<0.10	<0.10	<0.10	<0.10	<0.10	<0.10	<0.10	<0.10	<0.10	<0.10
PFDS	<0.03	<0.03	<0.03	<0.03	<0.03	<0.03	<0.03	<0.03	<0.03	<0.03	<0.03	<0.03
PFDoDS	<0.03	<0.03	<0.03	<0.03	<0.03	<0.03	<0.03	<0.03	<0.03	<0.03	<0.03	<0.03

Table S11. PFAA concentration (ng/L) and flow rate (m^3/s) in streams 13-15 during the sample collection from March to December 2019.

Stream	13				14				15			
Sample location	Munksjön				Orrnäsaån				Röttleån			
Sampling month	March	June	September	December	March	June	September	December	March	June	September	December
Flow rate m^3/s	5.560	2.310	2.570	7.270	1.100	0.367	0.053	0.781	0.593	0.296	0.037	0.875
TFA	63	60	77	80	140	180	180	220	170	170	140	200
PFPrA	0.66	1.5	0.88	0.74	0.64	1.7	1.1	0.70	<0.60	0.91	0.87	<0.60
PFBA	1.1	1.7	2.1	2.3	1.5	3.1	5.5	2.4	<0.10	2.7	2.0	0.66
PFPeA	1.5	3.3	<0.07	<0.07	<0.07	<0.07	1.0	<0.07	<0.07	<0.07	<0.07	<0.07
PFHxA	<0.25	2.2	1.9	<0.25	<0.25	<0.25	1.0	<0.25	<0.25	<0.25	0.49	<0.25
PFHpA	1.2	1.3	1.7	1.9	<0.03	<0.03	0.84	<0.03	<0.03	0.53	<0.03	<0.03
PFOA	3	2.7	3.3	3.5	1.5	2.0	3.4	3.1	1.3	3.3	2.8	2.4
PFNA	<0.03	0.30	0.36	0.35	<0.03	0.42	0.58	<0.03	0.12	0.40	0.26	<0.03
PFDA	<0.04	0.15	0.14	<0.04	<0.04	<0.04	0.29	<0.04	<0.04	0.14	<0.04	<0.04
PFUnDA	<0.10	<0.10	<0.10	<0.10	<0.10	<0.10	<0.10	<0.10	<0.10	<0.10	<0.10	<0.10
PFDoDA	1.5	<0.03	<0.03	<0.03	0.11	<0.03	<0.03	<0.03	<0.03	<0.03	<0.03	<0.03
TFMS	0.69	0.93	0.60	<0.10	0.86	0.99	0.78	1.3	0.87	0.72	0.85	0.86
PFEtS	<0.24	<0.24	<0.24	<0.24	0.33	0.29	0.27	0.31	0.34	0.29	0.27	<0.24
PFPrS	0.45	<0.10	<0.10	<0.10	0.47	<0.10	<0.10	0.49	0.47	<0.10	<0.10	0.45
PFBS	0.63	0.70	0.83	0.66	0.42	0.47	0.70	0.43	0.29	0.26	0.39	<0.10
PFPeS	<0.03	0.15	0.24	<0.03	<0.03	<0.03	<0.03	<0.03	<0.03	<0.03	<0.03	<0.03
PFHxS	1.1	0.94	1.2	1.5	<0.03	<0.03	0.22	0.41	0.14	<0.03	0.14	0.19
PFHpS	<0.10	<0.10	<0.10	<0.10	<0.10	<0.10	<0.10	<0.10	<0.10	<0.10	<0.10	<0.10
PFOS	3.7	2.0	2.2	3.8	0.47	0.21	<0.15	<0.15	0.24	0.50	<0.15	0.16
PFNS	<0.10	<0.10	<0.10	<0.10	<0.10	<0.10	<0.10	<0.10	<0.10	<0.10	<0.10	<0.10
PFDS	<0.03	<0.03	<0.03	<0.03	<0.03	<0.03	<0.03	<0.03	<0.03	<0.03	<0.03	<0.03
PFDoDS	<0.03	<0.03	<0.03	<0.03	<0.03	<0.03	<0.03	<0.03	<0.03	<0.03	<0.03	<0.03

Table S12. PFAA concentration (ng/L) and flow rate (m^3/s) in streams 16-18 during the sample collection from March to December 2019.

Stream	16				17				18			
Sample location	Svedån				Ålebäcken				Sandserydsån			
Sampling month	March	June	September	December	March	June	September	December	March	June	September	December
Flow rate m^3/s	0.788	0.270	0.237	0.673	1.030	0.895	0.031	1.460	0.203	0.011	0.028	0.367
TFA	53	39	49	69	660	260	130	820	66	98	110	85
PFPrA	<0.60	<0.60	<0.60	<0.60	0.68	0.66	<0.60	<0.60	0.92	1.9	1.3	1.1
PFBA	0.56	<0.10	<0.10	0.67	0.37	2.5	1.1	1.0	2.3	3.6	4.5	4.2
PFPeA	<0.07	1.1	<0.07	<0.07	<0.07	<0.07	<0.07	<0.07	8.8	14	9.8	6.9
PFHxA	<0.25	<0.25	<0.25	<0.25	<0.25	<0.25	<0.25	<0.25	11	9.7	8.7	9.5
PFHpA	<0.03	<0.03	<0.03	<0.03	<0.03	<0.03	<0.03	<0.03	8.5	9.6	6.1	10
PFOA	1.3	2.6	2.5	2.8	0.90	2.8	2.2	2.5	7.4	8.1	5.8	6.5
PFNA	0.17	<0.03	<0.03	<0.03	<0.03	<0.03	<0.03	<0.03	0.98	1.0	1.3	1.2
PFDA	<0.04	0.07	<0.04	<0.04	<0.04	<0.04	<0.04	<0.04	0.15	0.11	<0.04	<0.04
PFUnDA	<0.10	<0.10	<0.10	<0.10	<0.10	<0.10	<0.10	<0.10	0.12	<0.10	<0.10	<0.10
PFDoDA	0.04	<0.03	<0.03	<0.03	0.08	<0.03	<0.03	<0.03	<0.03	<0.03	<0.03	<0.03
TFMS	0.25	0.74	0.27	1.6	0.57	0.67	<0.10	0.59	0.25	1.3	0.74	0.25
PFEtS	0.36	<0.24	<0.24	<0.24	0.62	0.25	<0.24	0.41	0.27	<0.24	<0.24	0.25
PFPrS	<0.10	0.44	<0.10	<0.10	<0.10	<0.10	<0.10	0.47	0.59	<0.10	0.53	0.56
PFBS	0.10	<0.10	<0.10	<0.10	0.18	<0.10	0.15	<0.10	2.0	1.0	1.4	2.0
PFPeS	<0.03	<0.03	<0.03	<0.03	<0.03	<0.03	<0.03	<0.03	2.0	0.74	0.85	1.7
PFHxS	<0.03	<0.03	<0.03	0.09	0.14	0.13	0.08	0.14	12	6.0	5.5	11
PFHpS	<0.10	<0.10	<0.10	<0.10	<0.10	<0.10	<0.10	<0.10	0.87	0.40	0.32	0.95
PFOS	0.37	<0.15	<0.15	<0.15	0.17	0.20	<0.15	<0.15	38	27	17	51
PFNS	<0.10	<0.10	<0.10	<0.10	<0.10	<0.10	<0.10	<0.10	<0.10	<0.10	<0.10	<0.10
PFDS	<0.03	<0.03	<0.03	<0.03	<0.03	<0.03	<0.03	<0.03	<0.03	<0.03	<0.03	<0.03
PFDoDS	<0.03	<0.03	<0.03	<0.03	<0.03	<0.03	<0.03	<0.03	<0.03	<0.03	<0.03	<0.03

Table S13. PFAA concentration (ng/L) and flow rate (m^3/s) in streams 19 (outflow) and 20 (reference) during the sample collection from March to December 2019.

Stream	19				20			
Sample location	Motala ström				Unden			
Sampling month	March	June	September	December	March	June	September	December
Flow rate m^3/s	19	19	29	28	NA	NA	NA	NA
TFA	41	42	30	49	40	44	48	42
PFPrA	0.68	0.94	0.68	0.63	0.61	0.82	0.61	0.62
PFBA	<0.10	0.75	2.3	0.50	0.85	0.75	1.3	1.2
PFPeA	<0.07	0.96	<0.07	<0.07	<0.07	0.33	<0.07	<0.07
PFHxA	0.51	0.68	0.67	0.54	<0.25	0.26	<0.25	<0.25
PFHpA	0.48	0.42	0.39	0.38	0.38	0.39	0.44	0.39
PFOA	2.0	2.8	3.2	2.8	2.6	1.8	3.5	2.5
PFNA	0.22	0.19	0.40	<0.03	<0.03	0.32	0.34	0.23
PFDA	<0.04	0.10	<0.04	<0.04	0.14	0.10	0.07	<0.04
PFUnDA	<0.10	<0.10	<0.10	<0.10	<0.10	<0.10	<0.10	0.10
PFDoDA	0.15	<0.03	<0.03	<0.03	<0.03	<0.03	<0.03	<0.03
TFMS	0.20	0.35	0.35	0.64	1.9	0.45	0.20	0.11
PFEtS	0.29	<0.24	<0.24	<0.24	<0.24	<0.24	<0.24	<0.24
PFPrS	0.46	<0.10	0.47	0.46	<0.10	<0.10	<0.10	<0.10
PFBS	0.23	0.28	0.28	0.21	0.14	0.10	0.12	0.13
PFPeS	0.15	0.09	0.12	<0.03	<0.03	<0.03	<0.03	<0.03
PFHxS	0.70	0.63	0.71	0.60	0.10	0.10	0.09	<0.03
PFHpS	<0.10	<0.10	<0.10	<0.10	<0.10	<0.10	<0.10	<0.10
PFOS	0.77	0.68	0.76	0.92	18	0.38	0.30	<0.15
PFNS	<0.10	<0.10	<0.10	<0.10	<0.10	<0.10	<0.10	<0.10
PFDS	<0.02	<0.02	<0.02	<0.02	<0.03	<0.03	<0.03	<0.03
PFDoDS	<0.02	<0.02	<0.02	<0.02	<0.03	<0.03	<0.03	<0.03

Table S14. PFAA concentration (ng/L) and flow rate (m^3/s) in effluent from the sewage treatment plant and the paper mill during the sample collection from March 2019 to December 2019.

Sample location	Sewage treatment plant				Paper mill			
Sampling date	March	July	September	November	April	June	September	December
Flow rate m^3/s	0.020	0.017	0.015	0.027	0.304	0.318	0.306	0.289
TFA	150	160	110	200	41	51	33	39
PFPrA	0.93	1.1	1.1	0.92	<0.60	<0.60	0.67	<0.60
PFBA	2.0	3.8	3.6	2.4	<0.10	<0.10	<0.10	0.58
PFPeA	5.7	7.6	5.9	5.4	<0.07	<0.07	<0.07	<0.08
PFHxA	5.0	6.9	4.2	4.9	8.1	<0.25	<0.26	<0.27
PFHpA	2.4	2.5	1.6	2.9	<0.03	<0.04	<0.05	<0.06
PFOA	5.4	7.1	6.0	5.5	2.5	2.6	3.0	2.9
PFNA	0.04	1.1	0.87	0.69	0.19	0.68	0.21	<0.03
PFDA	0.28	0.64	0.65	<0.04	<0.04	<0.04	<0.04	<0.04
PFUnDA	<0.10	<0.10	<0.10	<0.04	<0.10	<0.10	<0.10	<0.10
PFDoDA	<0.03	<0.03	<0.03	<0.03	<0.03	<0.03	<0.03	<0.03
TFMS	1.4	2.2	1.6	1.3	<0.10	0.43	0.38	0.26
PFEtS	<0.24	<0.24	<0.24	0.26	<0.24	0.26	<0.24	<0.24
PFPrS	0.50	<0.10	<0.10	<0.10	<0.10	<0.10	0.46	<0.10
PFBS	1.5	4.2	2.6	2.5	<0.10	<0.11	<0.12	<0.13
PFPeS	0.16	0.23	0.12	<0.03	<0.03	<0.03	<0.03	<0.03
PFHxS	1.3	1.0	0.78	1.7	0.58	<0.03	0.61	<0.03
PFHpS	<0.10	<0.10	<0.10	<0.10	<0.10	<0.10	<0.10	<0.10
PFOS	1.1	1.8	1.6	0.98	0.60	<0.15	0.52	0.43
PFNS	<0.10	<0.10	<0.10	<0.10	<0.10	<0.10	<0.10	<0.10
PFDS	<0.03	<0.03	<0.03	<0.03	<0.03	<0.03	<0.03	<0.03
PFDoDS	<0.03	<0.03	<0.03	<0.03	<0.03	<0.03	<0.03	<0.03

Table S15. PFAA concentration (ng/L) in surface water from two locations in Lake Vättern (A and B) at 0.5 m during spring when the water column is mixed and at 0.5 m and 30 m during summer when the water column is stratified. The samples were collected in 2019.

Sample location	A	B	A	B	A	B
Sampling depth (m)	0.5	0.5	0.5	0.5	30	30
Sampling month	April	April	July	August	July	August
TFA	31	31	44	32	31	35
PFPrA	0.45	0.47	0.53	0.54	0.46	0.52
PFBA	0.42	0.38	0.68	0.64	0.70	0.55
PFPeA	<0.04	0.50	0.44	0.44	0.43	0.42
PFHxA	0.74	0.57	0.56	0.57	0.56	0.56
PFHpA	0.40	0.40	0.41	0.41	0.37	0.41
PFOA	1.9	1.8	2.0	2.0	2.0	2.0
PFNA	0.22	0.18	0.19	0.20	0.20	0.20
PFDA	0.03	<0.02	0.04	0.04	0.03	0.03
PFUnDA	<0.05	<0.05	<0.05	<0.05	<0.05	<0.05
PFDoDA	0.05	0.03	<0.01	<0.01	<0.01	<0.01
TFMS	0.18	0.21	0.36	0.31	0.25	0.22
PFEtS	<0.12	<0.12	<0.12	<0.12	<0.12	<0.12
PFPrS	0.22	<0.05	0.24	<0.05	<0.05	0.24
PFBS	<0.05	0.23	0.25	0.24	0.23	0.21
PFPeS	0.10	0.13	0.13	0.12	0.13	0.13
PFHxS	0.84	0.62	0.67	0.65	0.70	0.68
PFHpS	<0.05	<0.05	<0.05	<0.05	<0.05	<0.05
PFOS	0.57	0.76	0.60	0.61	0.60	0.63
PFNS	<0.05	<0.05	<0.05	<0.05	<0.05	<0.05
PFDS	<0.01	<0.01	<0.01	<0.01	<0.01	<0.01
PFDoDS	<0.01	<0.01	<0.01	<0.01	<0.01	<0.01

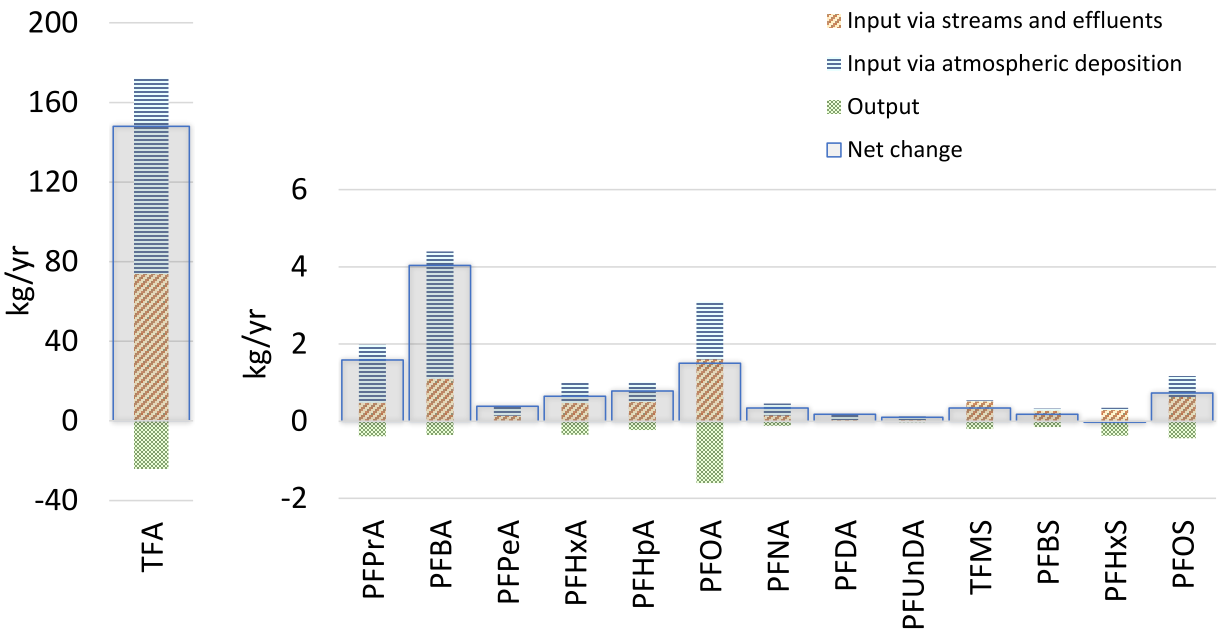

Figure S2. Net change (kg/yr) of PFAAs in Lake Vättern during the sampling period in 2019–2020 because of input via streams, effluents and atmospheric deposition and output via streams.

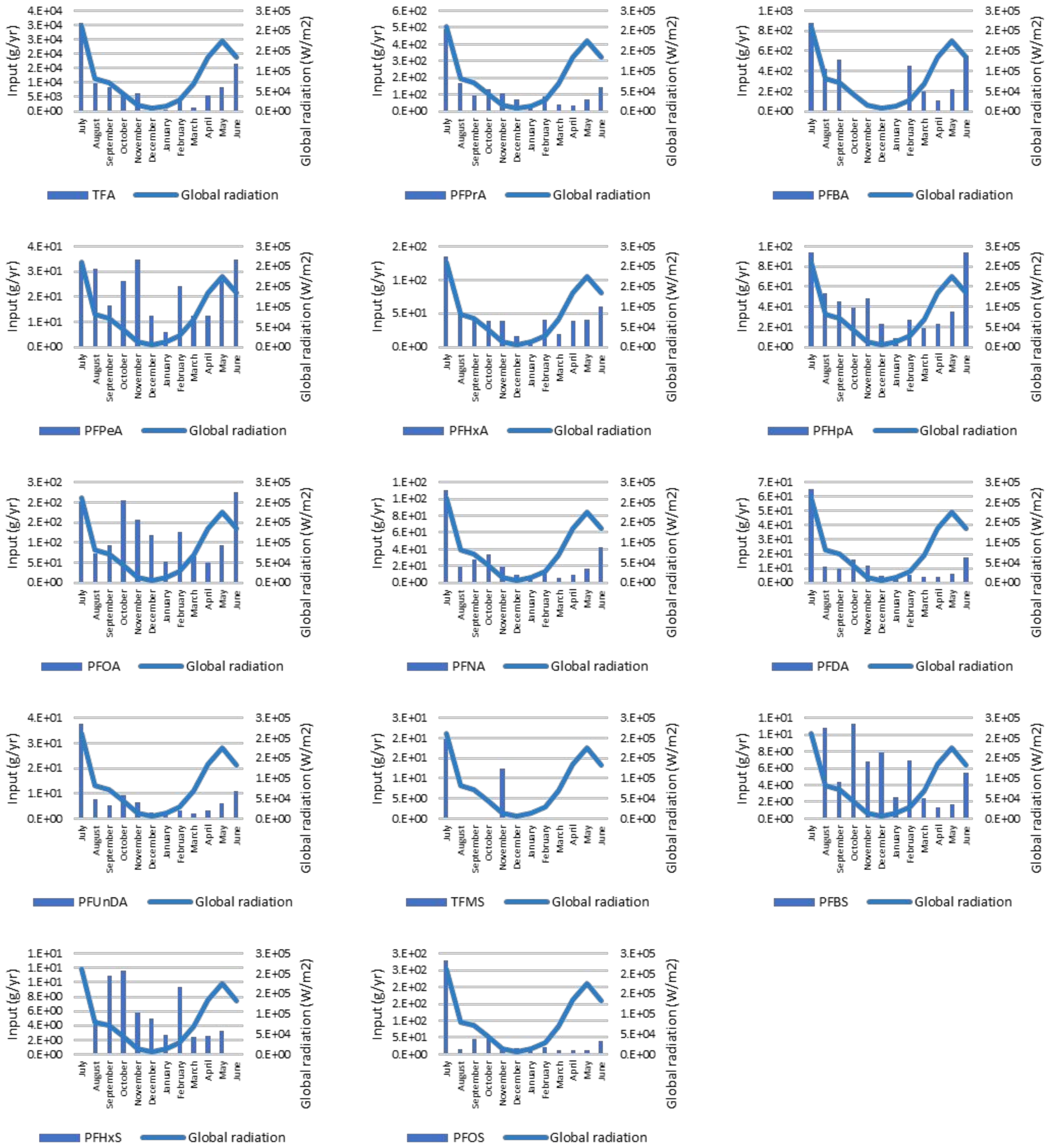

Figure S3. PFAA input from atmospheric deposition (g/yr) and global radiation (W/m^2).

References

1. SMHI (Swedish Meteorological and Hydrological Institute). Fakta om Vättern. https://www.smhi.se/kunskapsbanken/hydrologi/de-storasjoarna/fakta-om-vattern-1.4730 (accessed 2021-08-06).
